# The role of patiromer: Comparing OPAL-HK data with untreated real-world patients in the United Kingdom—A retrospective, propensity-matched analysis

**DOI:** 10.1371/journal.pone.0237467

**Published:** 2020-08-27

**Authors:** Ibrahim Ali, Rajkumar Chinnadurai, Georgiana Cornea, Michele Intorcia, Philip A. Kalra

**Affiliations:** 1 Department of Renal Medicine, Salford Royal NHS Foundation Trust, Salford, United Kingdom; 2 Vifor Pharma Group, Glattbrugg, Switzerland; University Medical Center Utrecht, NETHERLANDS

## Abstract

**Objectives:**

The first phase of the published OPAL-HK study was a single-group treatment phase, which showed that patiromer normalised serum potassium at 4weeks in patients with chronic kidney disease stages 3–4 who were receiving renin-angiotensin-aldosterone inhibitors. We utilised real-world data to provide a control comparison to evaluate patiromer’s efficacy in lowering serum potassium.

**Materials and methods:**

The Salford Kidney Study (SKS) in the United Kingdom provided a matched cohort. After applying OPAL-HK inclusion and exclusion criteria, patients with an outpatient potassium level between 5.1mmol/L to <6.5mmol/L and whose next outpatient level was checked 24–42 days later were selected. Patients underwent 1:1 matching with the 243 OPAL-HK patients using propensity matching based on 6 variables: age, gender, estimated glomerular filtration rate, diabetes, heart failure and potassium level. The study outcomes aligned with the OPAL-HK treatment phase: mean change in baseline potassium, and the proportion of patients with a potassium of 3.8 to <5.1mmol/L at follow-up.

**Results:**

The study comprised 87 precisely matched patients. The mean follow-up in the 87 SKS patients was 31±5 days. At baseline, matched patients had a mean potassium of 5.5±0.3mmol/L. At follow-up, the mean level was unchanged in SKS patients but was 4.5±0.5mmol/L in the OPAL-HK group (p<0.001), a mean (±SE) change of -1.00±0.06mmol/L. The target range of 3.8 to <5.1mmol/L was reached in 80% of OPAL-HK patients compared with 0% in the SKS cohort. There were very few interventions undertaken to reduce hyperkalaemia in SKS patients.

**Conclusions:**

Using real-world data as a matched control arm for the first phase of the OPAL-HK study, we highlight a potential role for patiromer in lowering potassium levels in patients with CKD 3–4 receiving renin-angiotensin-aldosterone inhibitors.

## Introduction

Hyperkalaemia is an important electrolyte disturbance that most commonly occurs in patients with advanced chronic kidney disease (CKD) [1]. Notwithstanding the potentially life-threatening cardiac arrhythmias that can arise with hyperkalaemia, patients also face the potential undesirable consequence of reducing or indefinitely discontinuing renin-angiotensin-aldosterone system inhibitors (RAASi), which are known to provide long-term reno- and cardioprotection [2]. Therefore, efforts to maintain normokalaemia and permit continuation of RAASi is a well-established tenet for optimal CKD management [3]. Achieving normokalaemia in the outpatient setting whilst maintaining RAASi may be achieved through a combination of measures including low-potassium dietary advice, addition of a loop or thiazide diuretic, correction of acidosis with sodium bicarbonate and with potassium binders such as sodium polystyrene sulfonate (SPS). This latter intervention, beset by a lack of evidence for long-term use and the significant risk of gastrointestinal side effects [4], has been transformed thanks to the introduction of new oral potassium-binding agents such as patiromer. Patiromer is a non-absorbed, sodium-free potassium-binding polymer that non-specifically binds potassium for calcium along the gastrointestinal tract, facilitating potassium excretion.

OPAL-HK is a major multicentre prospective trial that investigated the efficacy and safety of patiromer at lowering potassium levels in CKD patients [5]. The study recruited 243 patients with CKD stages 3–4 who were receiving RAASi and whose baseline serum potassium was 5.1 to <6.5mmol/L. The initial part of the study was a single-group, single-blinded treatment phase with patiromer over 4weeks, followed by a placebo-controlled withdrawal phase over 8 weeks. In the treatment phase, patiromer was shown to reduce serum potassium by a mean ± standard error (SE) of -1.01±0.03mmol/L, and 76% of patients reached a target potassium range of 3.8 to <5.1mmol/L at the end of the 4week follow-up. In this phase, serum potassium levels were measured at baseline and on day 3 and weekly thereafter. In the withdrawal phase, discontinuation of patiromer resulted in a statistically higher serum potassium and a higher proportion of patients with levels >5.5mmol/L at the end of follow-up compared to the group that continued to take patiromer.

Given the lack of a control arm for the first phase of the OPAL-HK trial, we undertook a study to provide further insight into the efficacy of patiromer. The aims were to (1) utilise real-world data from an observational CKD cohort in the United Kingdom (UK) to provide a control, untreated, comparison group to the first phase of OPAL-HK and evaluate patiromer’s efficacy in lowering serum potassium; and (2) demonstrate the feasibility of comparing real-world patient data with clinical trial data, which is particularly pertinent to hyperkalaemia trials where placebo interventions would be deemed unethical.

## Materials and methods

### Patient population

Patients for the matched cohort were selected from the Salford Kidney Study (SKS). This is an ongoing prospective observational study in the UK that has been recruiting patients aged ≥18 years with CKD stage 3–5 since 2002. Demographic data is collected at entry into SKS. Blood and urine sampling for routine clinical tests is performed at baseline and at subsequent clinic visits and results are readily available on the hospital’s electronic patient record. Patients are followed in SKS until endpoints are reached, which include death, initiation of renal replacement therapy (chronic dialysis or transplantation), loss to follow-up, discharge from renal clinic or withdrawal of consent. The study complies with the declaration of Helsinki and ethical approval has been obtained from the regional ethical committee (current REC reference 15/NW/0818). All participants provided written informed consent.

### Creating a matched cohort

The first phase of OPAL-HK enrolled 243 patients aged 18–80 years with CKD stages 3–4, (corresponding to an estimated glomerular filtration rate (eGFR) of 15 to <60ml/min/1.73m^2^, calculated by either CKD-EPI or MDRD equation [5]), and whose baseline potassium was between 5.1 to <6.5mmol/L. All patients were receiving stable doses of RAASi for at least 28 days. Exclusion criteria in the OPAL-HK study included a high potassium requiring emergency treatment at baseline, type 1 diabetes, systolic blood pressure ≥180mmHg or <110mmHg or a diastolic blood pressure of ≥110mmHg or <60mmHg, use of potassium-altering chronic medications if doses not stable 28 days prior to selection (loop and thiazide diuretics, non-selective beta blockers, amiloride, triamterene, drospirenone, non-steroidal anti-inflammatory drugs, cyclooxygenase-2 inhibitors, digoxin, bronchodilators, theophylline, heparin, synthetic thyroid hormone) and current use of sodium bicarbonate, sodium polystyrene sulfonate, calcium polystyrene sulfonate and potassium supplements. Patients were followed-up for 4 weeks.

**Fig 1:**
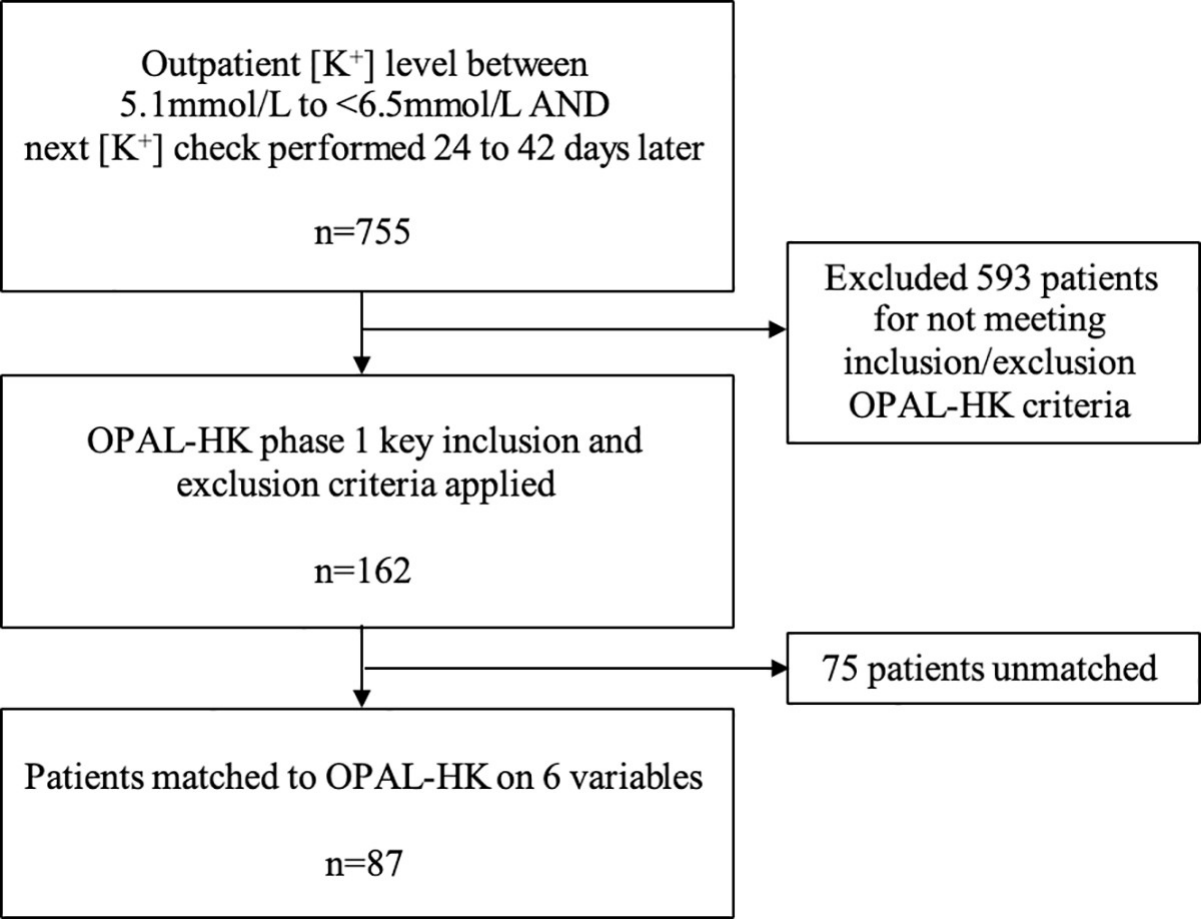
Patient selection from the Salford kidney study.

To acquire a matched cohort to the 243 OPAL-HK patients, a three-step process was applied to patient selection in SKS (Fig 1). First, patients were chosen if they had an outpatient potassium level at any point after recruitment into SKS between 5.1mmol/L and <6.5mmol/L and whose next outpatient potassium level was obtained 24 to 42 days (3.5 to 6 weeks) later. This follow-up timeframe was chosen, as opposed to a precise 28 days, to account for the spread of clinic availability in real-world practice. Secondly, key OPAL-HK inclusion and exclusion criteria as listed above were applied. All criteria were cross-checked by reviewing patient’s clinic letters and drug prescriptions from the hospital electronic health record. This provided a patient cohort of 162 patients. Baseline demographic data for these patients were updated to reflect the time-point of entry into this analysis. The final stage involved 1:1 matching of 162 SKS patients with 243 OPAL-HK patients using propensity scores based on 6 baseline variables: age, gender, eGFR, diabetes, heart failure and potassium level. This step resulted in a final cohort of 87 SKS patients matched to 87 partner patients in OPAL-HK. All 87 patients in the OPAL-HK cohort had completed the full 4 weeks of patiromer treatment.

### Study endpoints in the matched analysis

The study endpoints for this analysis were aligned to those in the first phase of the OPAL-HK study: the primary endpoint was the mean change in serum potassium from baseline to follow-up, and the secondary endpoint was the proportion of patients who had a serum potassium in the range of 3.8 to <5.1mmol/L at follow-up.

### Statistical analysis

Propensity scores were generated using binary logistic regression that utilised six baseline variables described above. Patients were matched in a 1:1 ratio using the nearest neighbour method with the same propensity score (S1 Fig).

Continuous data is presented as means ± standard deviation (SD) and categorical data expressed as total numbers with percentages. Baseline differences between SKS and OPAL-HK groups were analysed with independent Student’s t-test for continuous data and Chi-squared tests for categorical data. Comparison between eGFR and potassium results in SKS patients at baseline and follow-up were analysed using paired t-test. A p-value of <0.05 was considered significant. All analyses including propensity score matching was performed using IBM SPSS (Version 22), licensed to University of Manchester.

## Results

### Baseline characteristics

Table 1 highlights the baseline characteristics of the SKS cohort that met the inclusion and exclusion criteria of the first phase of the OPAL-HK study. There were similarities between the two groups with respect to age and gender, but they were statistically dissimilar with respect to baseline potassium level, eGFR and the presence of diabetes and heart failure. A precisely matched cohort of 87 patients in each patient group was subsequently created based on 6 variables (Table 2). The matched cohorts were used for analysis of study endpoints.

**Table 1:** Comparison of baseline characteristics between patients in the first phase of the OPAL-HK study and SKS patients meeting the inclusion and exclusion criteria of the study.

Baseline characteristic	OPAL-HK (n = 243)	SKS (n = 162)	P-value
Male sex–no. (%)	140 (58)	90 (56)	0.709
Age–years	64.2 ± 10.5	63.2 ± 13.4	0.423
White race–no. (%)	239 (98)	159 (98)	0.876
Type 2 diabetes–no. (%)	139 (57)	67 (41)	0.002
Heart failure–no. (%)	104 (42)	40 (25)	<0.001
Myocardial infarction–no. (%)	60 (25)	8 (5)	<0.001
Hypertension–no. (%)	236 (97)	162 (100)	0.029
Serum [K^+^] (mmol/L)	5.6 ± 0.5	5.4 ± 0.3	0.002
eGFR (ml/min/1.73m^2^)	35.4 ± 16.2	29.8 ± 10.7	<0.001
RAASi use			
• ACEi–no. (%)	170 (70)	108 (67)	0.569
• ARB–no. (%)	92 (38)	70 (43)	0.231
• Aldosterone antagonist–no. (%)	22 (9)	8 (5)	0.122
• Renin inhibitor–no. (%)	2 (1)	2 (1)	0.682
• Dual-blockade* –no. (%)	41 (17)	32 (20)	0.373
Diuretic use–no. (%)	132 (54)	85 (52)	0.807

Continuous data is presented as means (±SD). P value by Student’s t test for continuous data and chi-squared test for categorial data.

**Abbreviations**: eGFR: estimated glomerular filtration rate, calculated using CKD-EPI in SKS patients; ACEi: angiotensin-converting enzyme inhibitor; ARB: angiotensin receptor blocker.

*Dual RAAS blockade indicates any combination of ≥2 the following: ACEi, ARB, aldosterone antagonist, or renin inhibitor.

**Table 2:** Comparison of baseline characteristics in the matched cohorts.

Baseline characteristic	OPAL-HK (n = 87)	SKS (n = 87)	P-value
Male sex–no. (%)	47 (54)	52 (60)	0.445
Age–years	63.7 ± 9.5	63.9 ± 13.3	0.934
Type 2 diabetes–no. (%)	46 (53)	45 (52)	0.880
Heart failure–no. (%)	24 (28)	29 (33)	0.412
Serum [K^+^] (mmol/L)	5.5 ± 0.3	5.5 ± 0.3	0.678
eGFR (ml/min/1.73m^2^)	31.2 ± 11.7	30.9 ± 11.9	0.873

P value by Student’s t test and chi-squared test for categorical data.

**Abbreviations**: eGFR: estimated glomerular filtration rate, calculated using CKD-EPI in SKS patients.

### Study endpoints in the matched analysis

**Fig 2:**
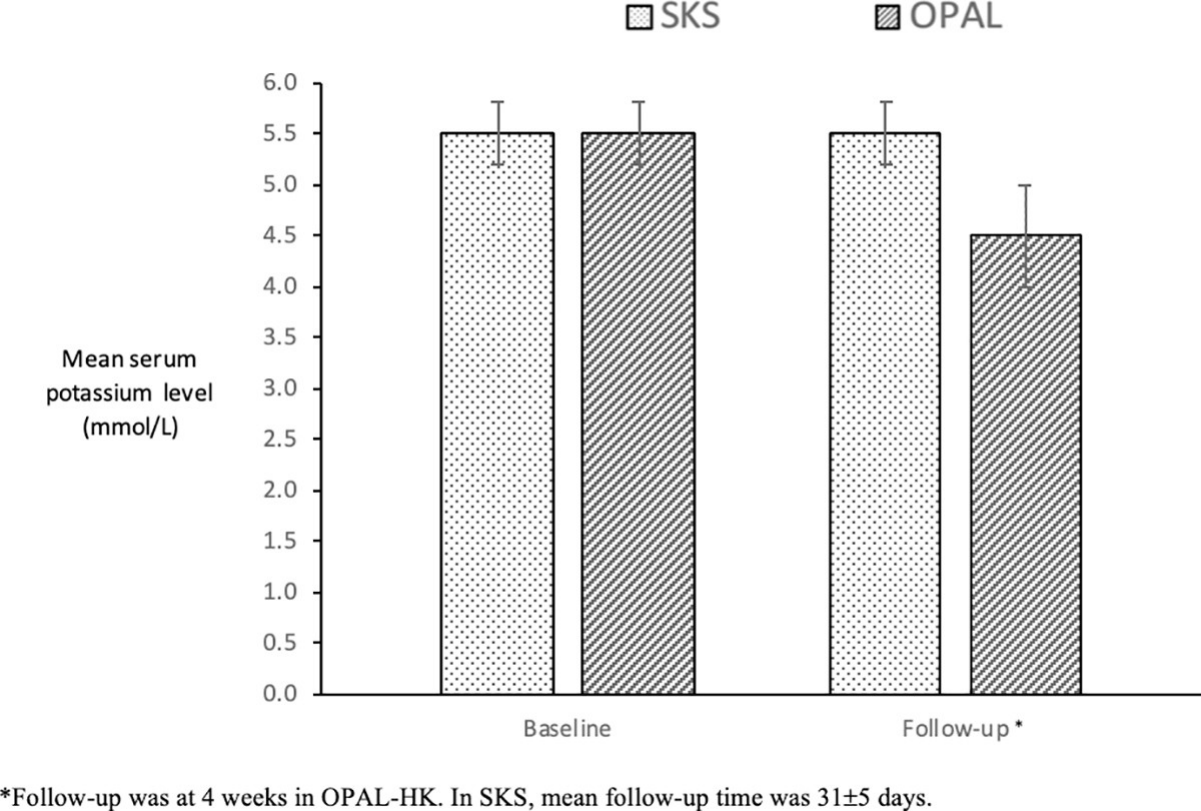
Change in mean potassium level from baseline to follow-up. * Follow-up was at 4 weeks in OPAL-HK. In SKS, mean follow-up time was 31±5 days.

The mean follow-up in the matched SKS cohort was 31±5 days. In both SKS and OPAL-HK patients, the mean baseline potassium level was identical at 5.5±0.3mmol/L. At the end of follow-up, the mean potassium level was unchanged in SKS patients but had significantly reduced to 4.5±0.5mmol/L in the OPAL-HK group (p<0.001) (Fig 2). This represented a mean (±SE) change of -1.00±0.06mmol/L, in line with the results of 243 patients within the OPAL-HK trial, which reported a mean (±SE) change of -1.01±0.03mmol/L. This change in potassium in OPAL-HK patients was a consistent feature observed in patients in different stages of CKD with or without diabetes and heart failure (Table 3).

**Table 3:** Changes in mean potassium from baseline to follow-up according to CKD stage, and presence of diabetes or heart failure.

	SKS			OPAL-HK		
	Patient numbers	Mean [K^+^] (mmol/L)		Patient numbers	Mean [K^+^] (mmol/L)	
		Baseline	Follow-up		Baseline	Follow-up
CKD stage 3	39	5.4 ± 0.3	5.4 ± 0.3	36	5.5 ± 0.3	4.5 ± 0.5
CKD stage 4	48	5.5 ± 0.3	5.5 ± 0.3	51	5.5 ± 0.3	4.5 ± 0.5
CKD 3 or 4 + diabetes only	29	5.6 ± 0.4	5.5 ± 0.4	33	5.5 ± 0.3	4.5 ± 0.4
CKD 3 or 4 + heart failure only	13	5.5 ± 0.3	5.3 ± 0.2	11	5.5 ± 0.2	4.5 ± 0.7
CKD 3 or 4 + diabetes + heart failure	16	5.5 ± 0.3	5.3 ± 0.2	13	5.5 ± 0.2	4.5 ± 0.7

[K^+^] values expressed as mean ± SD.

**Fig 3:**
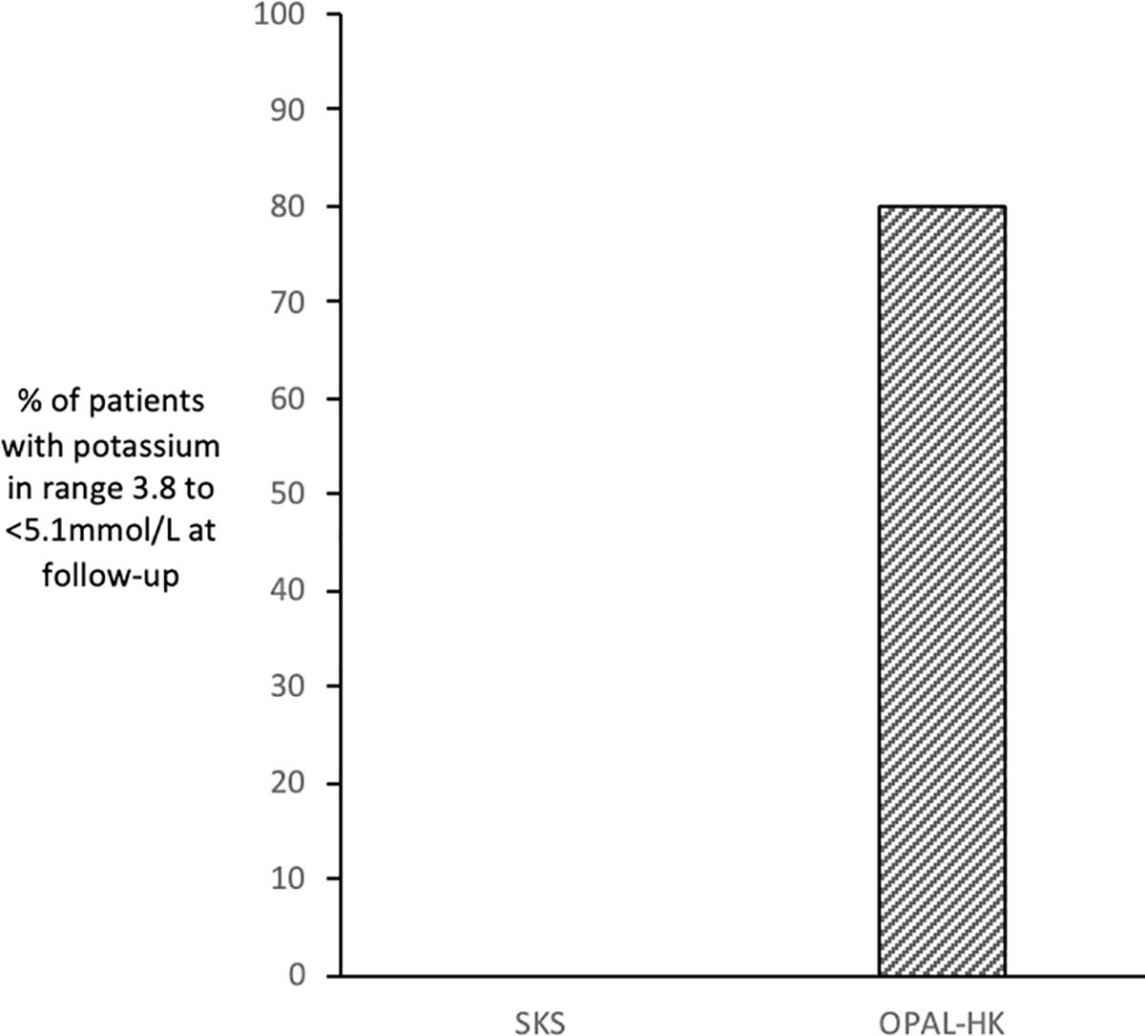
Proportion of patients in potassium range 3.8 to <5.1mmol/L at follow-up.

For the secondary endpoint, 80% of patients in OPAL-HK (70 of 87 patients) reached the target potassium range of 3.8 to <5.1mmol/L at the end of follow-up compared with 0% from the comparator SKS cohort (Fig 3). This is similar to the 76% of patients quoted to reach this target range in the published OPAL-HK study.

Just as in the OPAL-HK study, there was no clinically significant change in renal function from baseline to follow-up in the 87 matched SKS patients: mean baseline eGFR was 30.9±11.9ml/min/1.73m^2^ and 31.9±12.6ml/min/1.73m^2^ at follow-up (p = 0.144).

The number of patients that switched from one potassium range to another, from baseline to follow-up, are shown in Table 4, stratified across CKD stages. The majority of patients in OPAL-HK reached a potassium level of <5.1mmol/L irrespective of their baseline potassium range and CKD stage. In contrast, SKS patients were spread more heterogeneously across the range values at follow-up. For instance, 52 patients had a baseline potassium of 5.1–5.5mmol/L but only 39 patients remained in this range at follow-up; 13 patients had results in higher potassium ranges. At follow-up in the group of 29 patients with baseline potassium between 5.6–6.0mmol/L, 16 patients had a lower potassium, 9 remained in the same range and 4 had a higher potassium level.

**Table 4:** Baseline and follow-up potassium in SKS and OPAL-HK patients stratified to CKD stage.

			Follow-up [K^+^] range (mmol/L)			
			<5.1	5.1–5.5	5.6–6.0	>6.0
		Baseline [K^+^] range (mmol/L)	n (%)	n (%)	n (%)	n (%)
	**CKD 3 at baseline (n = 39)**	**5.1–5.5 (n = 28)**	0 (0)	21 (75)	4 (14)	3 (11)
		**5.6–6.0 (n = 10)**	0 (0)	8 (80)	1 (10)	1 (10)
**SKS patients**		**>6.0 (n = 1)**	0 (0)	0 (0)	1 (100)	0 (0)
	**CKD 4 at baseline (n = 48)**	**5.1–5.5 (n = 24)**	0 (0)	18 (75)	6 (25)	0 (0)
		**5.6–6.0 (n = 19)**	0 (0)	8 (42)	8 (42)	3 (16)
		**>6.0 (n = 5)**	0 (0)	2 (40)	2 (40)	1 (20)
	**CKD 3 at baseline (n = 36)**	**5.1–5.5 (n = 23)**	20 (87)	3 (13)	0 (0)	0 (0)
**OPAL patients**		**5.6–6.0 (n = 12)**	11 (92)	1 (8)	0 (0)	0 (0)
		**>6.0 (n = 1)**	1 (100)	0 (0)	0 (0)	0 (0)
	**CKD 4 at baseline (n = 51)**	**5.1–5.5 (n = 26)**	19 (73)	4 (15)	3 (12)	0 (0)
		**5.6–6.0 (n = 23)**	22 (96)	1 (4)	0 (0)	0 (0)
		**>6.0 (n = 2)**	2 (100)	0 (0)	0 (0)	0 (0)

### Interventions in SKS

There were no interventions for patients with a baseline potassium of <6.0mmol/L. Amongst the 9 patients with a potassium of 6.0mmol/L or more, only 5 patients received an outpatient intervention as determined from a clinic letter or clinical note found in the patient’s electronic health record. Each of these patients received care from a different physician and interventions were not consistent at specific potassium values (S1 Table). Amongst the 5 patients who had an intervention, there was a mean change in potassium of -0.3±0.4mmol/L, with 4 out of the 5 patients reaching a potassium level of <6.0mmol/L, and this included 2 patients who had a change in their RAASi therapy. There were 4 patients, however, without any documented action, in whom the mean potassium change was -0.4±0.5mmol/L at follow-up.

### Unmatched analysis

A separate analysis was undertaken for the original unmatched 162 SKS patients that met the inclusion and exclusion criteria of the OPAL-HK study. Similar changes in potassium were found: the baseline mean potassium level was 5.4±0.3mmol/L, which remained unchanged at follow-up, and 0% achieved a potassium in the range 3.8 to <5.1mmol/L.

## Discussion

Comparing a real-world CKD cohort precisely matched to the first phase of the OPAL-HK study, we have shown that patiromer plays a role in lowering potassium levels in patients who have CKD stages 3 or 4 and who are taking RAASi treatment.

Undertaking placebo-controlled trials in hyperkalaemic patients would be considered unethical given the potential for harm to patients with uncorrected elevations in potassium [6]. We believe our study is the first to provide a real-world comparison arm to overcome this challenge faced by trials studying interventions for hyperkalaemia.

In our analysis, patiromer reduced potassium levels by -1.01±0.03mmol/L, and 80% of patients were in a target range of 3.8 to <5.1mmol/L at week 4. This was in sharp contrast to the SKS cohort, in which some patients developed potassium levels of >6.0mmol/L at follow-up even when their baseline potassium was between 5.1–5.5mmol/L (Table 3). No patient, however, had a follow-up potassium of >6.0mmol/L in the OPAL-HK cohort.

It is important to note that the SKS cohort is based in the UK, where national guidelines advise altering or discontinuing RAASi in patients with CKD only when the potassium is >6.0mmol/L [6]. This is clearly reflected in the small number of patients who underwent an alteration in their RAASi (2 out of 9; S1 Table) and also explains why the majority of SKS patients in this analysis did not experience an overall mean change in potassium. In effect, therefore, the SKS cohort was akin to a placebo group in which the vast majority of patients received no discernible action to lower potassium, which is an accurate reflection of the standard care for managing chronic hyperkalaemia in CKD patients in the UK.

Typical interventions for hyperkalaemia management include reducing or stopping RAASi, promoting a low-potassium diet (usually with input from a dietician), initiating or up-titrating sodium bicarbonate to correct acidosis and initiating or up-titrating diuretics in the presence of fluid overload [7]. Some of these actions were only implemented in the small group of SKS patients with a baseline potassium ≥6.0mmol/L, but there was significant variation in the care afforded by different clinicians (S1 Table).

A significant weight of evidence exists from several studies highlighting the clinical efficacy of patiromer at permitting use of RAASi by preventing hyperkalaemia. In PEARL-HF [8], 105 patients with either chronic heart failure and a history of hyperkalaemia resulting in discontinuation of a RAASi and/or beta-adrenergic blocking agent, or CKD (defined as eGFR<60ml/min/1.72m^2^) were initiated on spironolactone 25mg/day and were randomised to a double-blind treatment with either patiromer 25.2g/day or placebo for 4 weeks.

Spironolactone, initiated at 25mg/day, was increased to 50mg/day on day 15 if potassium was ≤5.1mmol/L. Patiromer was shown to reduce serum potassium levels with a difference between groups of -0.45mmol/L (p<0.001), reduce the incidence of patients reaching a potassium of >5.5mmol/L (7.3% patiromer vs. 24.5% placebo; p = 0.015), and allow a higher proportion of patients to up-titrate spironolactone to 50mg/day (91% patiromer vs. 74% placebo; p = 0.019). However, only 50% of patients had CKD in this study and this was reflected in the high mean (±SD) baseline eGFR of 84 ±35ml/min/1.73m^2^ in the treatment group. In contrast, the AMETHYST-DN [9] study focused on 306 diabetic patients with more advanced CKD (65% had CKD stage 3; 22% had stage 4) and who were all receiving RAASi. Patients were stratified to either having mild (>5.0–5.5mmol/L) or moderate (>5.5 to <6.0mmol/L) hyperkalaemia and then randomised to different doses of patiromer. The study showed that patiromer was effective at lowering potassium after 4weeks of treatment, and this effect was sustained for up to 1year. However, it was an unblinded study without a control arm. The AMBER trial [10], in contrast, was a double-blind, placebo-controlled trial, although it did not focus on patients with baseline hyperkalaemia. This study enrolled 295 patients aged ≥18 years, with an eGFR of 25-45ml/min/1.73m^2^, a baseline potassium between 4.3 and 5.1mmol/L and resistant hypertension. The main endpoint showed a statistically higher proportion of patients who were receiving patiromer remained on spironolactone compared with those receiving placebo (86% versus 66% respectively) at 12 weeks follow-up.

In addition to its efficacy at achieving and maintaining normokalaemia, patiromer has been consistently shown to be well tolerated with a very good safety profile. The majority of adverse effects reported from trials are gastrointestinal-related, including constipation (6.2%), diarrhoea (3.0%), abdominal pain (2.9%) and flatulence (1.8%). These adverse reactions are typically only mild-to-moderate in nature [11]. Hypomagnesaemia (5.3%) is also a recognised complication [11], and although there is no evidence of an increased risk of cardiac arrhythmias associated with patiromer-related hypomagnesaemia, monitoring of levels is advised and consideration given to magnesium replacement if it arises.

### Strengths and limitations

Our work has a number of limitations. Firstly, although diet was not controlled, patients in the OPAL-HK study received counselling on maintaining a low-potassium diet at each visit during the trial regardless of their baseline potassium level. This was not the case in the real-world SKS cohort, in which dietetic input was routinely sought only when patients developed potassium levels of ≥6.0mmol/L. Amongst the 87 matched SKS patients, 13 had received dietetic review for low-potassium guidance in the past. During the study period, only 3 patients received dietetic advice (S1 Table), all in instances where the potassium level was ≥6.0mmol/L. As the two cohorts differed in dietary potassium intervention, changes in potassium in the first phase of the OPAL-HK can only therefore partly be attributed to patiromer use. Secondly, OPAL-HK patients had a blood test at day 3 and then weekly thereafter for 4 weeks. In contrast, the SKS cohort had a blood test at baseline and then at follow-up, which was 3.5 to 6 weeks between the two potassium readings. The difference in intensive monitoring and follow-up is, however, only significant in that it permitted patients in OPAL-HK to receive dietary counselling at each visit. The less intensive and wider follow-up timeframe in the SKS cohort is a reflection of real-world practice, dependent upon clinical need as adjudged by the managing clinician. Thirdly, use of retrospective real-world data can be affected by a lack of quality in data recording. We attempted to overcome this by cross-checking the data to ensure all patients’ parameters were accurate at the point of entry into the analysis. We could not, however, account for changes in patient care beyond what had been recorded in clinic letters between the baseline and follow-up potassium measurement. Some SKS patients appeared to not receive any intervention despite their potassium being >6.0mmol/L but they may have received undocumented intervention. Given SKS patient selection in this study spanned the timeframe from the cohort’s inception in 2002, different patients may have been exposed to a differential degree of care. However, routine interventions for hyperkalaemia management has remained unchanged since 2002, and the 87 SKS patients in our analysis represent standards of care which is characteristic of current UK guidance and practice. Fourthly, given this work only focuses on UK patients, the results may not be generalisable to other patient populations. Finally, the total number of SKS patients that underwent analysis was much smaller than the 243 patients initially enrolled into OPAL-HK. The final groups, however, were precisely matched, and this is arguably a strength of the study as it permitted more robust analysis. Regardless, similar findings were reached in the unmatched cohort of 162 patients. In addition, given there was little difference in the results of the primary and secondary endpoints in our work compared to the OPAL-HK study, our 87 OPAL-HK patients were representative of the overall 243 patients in the OPAL-HK trial.

Notwithstanding the discrepancy in dietary intervention, the routine, real-world care in the SKS cohort still affords a valuable control arm in this specific study because (1) all patients met the inclusion and exclusion criteria of the OPAL-HK study, which excluded patients taking sodium bicarbonate or other potassium binders; (2) patients were closely matched based on propensity-matched scores; (3) there was no significant difference between baseline and follow-up eGFR and thus changes in renal function were unlikely to affect potassium levels; and (4) there were a minimal number of interventions in the SKS cohort, and thus the RAASi doses were unchanged in the vast majority of patients at follow-up.

## Conclusion

Achieving normokalaemia to permit continuation of prognostically beneficial RAASi remains a primary concern for optimal CKD care and for patients with heart failure. We provide evidence using a comparative UK-based CKD cohort that emphasises the potential benefit of patiromer in lowering potassium levels in patients with CKD stages 3–4 after 4 weeks of follow-up, whereas there was no change in potassium concentration in their real-world matched comparators, reflecting a minimal degree of potassium lowering interventions in regular clinical practice, especially in patients with levels above the normal range but less than 6.0mmol/L. Further longer-term prospective data evaluating patiromer against standard care in managing chronic hyperkalaemia, especially in patients with potassium levels >6.0mmol/L, and ideally enabling access to major clinical outcome data, would be desirable.

## Supporting information

S1 Fig Graphs to show balanced propensity scores between SKS and OPAL-HK patients after matching. (TIF)

S1 Table Interventions for SKS patients with baseline [K^+^] ≥6.0mmol/L. (PDF)

S1 Dataset Dataset for 87 matched patients. (XLSX)
