# Supplementary figures and images for: The role of patiromer: Comparing OPAL-HK data with untreated real-world patients in the United Kingdom—A retrospective, propensity-matched analysis
Source: PLoS One. 2020 Aug 27;15(8):e0237467. doi: 10.1371/journal.pone.0237467 (PMC7451519; doi:10.1371/journal.pone.0237467)

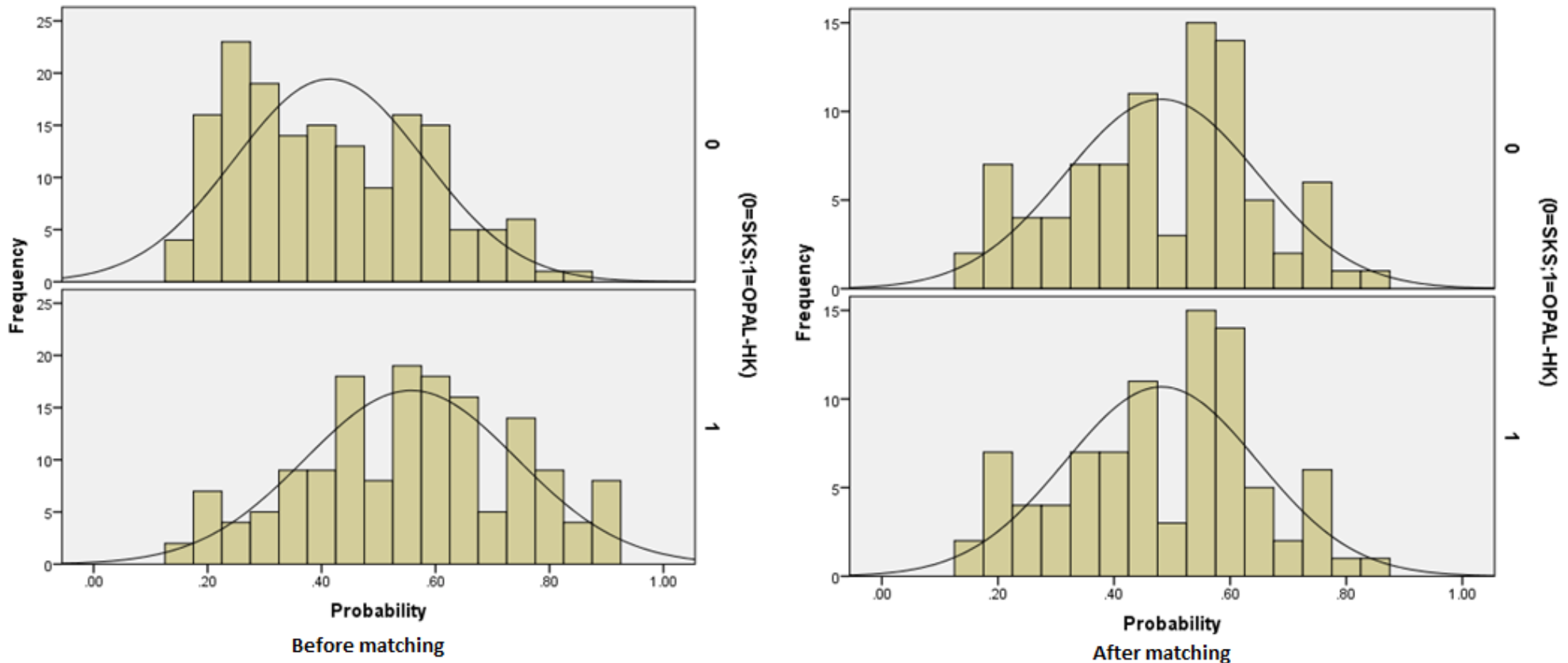

Supplement: S1 Fig — (TIF) [file pone.0237467.s001.tif]
