# Supplementary material for: The role of patiromer: Comparing OPAL-HK data with untreated real-world patients in the United Kingdom—A retrospective, propensity-matched analysis
Source: PLoS One. 2020 Aug 27;15(8):e0237467. doi: 10.1371/journal.pone.0237467 (PMC7451519; doi:10.1371/journal.pone.0237467)
Supplement: S1 Table — (PDF) [file pone.0237467.s002.pdf]

S1 Table. Interventions for SKS patients with baseline K^+≥6.0mmol/L

	Baseline $[K^+]$/mmol/L	Intervention	Follow-up $[K^+]$/mmol/L
**Patient 1**	6.0	No action	6.1
**Patient 2**	6.0	Dietary advice	6.3
**Patient 3**	6.0	ACEi stopped	5.6
**Patient 4**	6.1	Dietary advice	5.7
**Patient 5**	6.1	No action	6.1
**Patient 6**	6.2	Furosemide added	5.8
**Patient 7**	6.2	Dietary advice. ACEi halved	5.6
**Patient 8**	6.2	No action	5.4
**Patient 9**	6.4	No action	5.4

Abbreviations: ACEi (angiotensin converting enzyme inhibitor)
